# Studies on Gynogenesis Induction in Cassava (*Manihot esculenta* Crantz) Unpollinated Ovule Culture

**DOI:** 10.3389/fpls.2020.00365

**Published:** 2020-04-03

**Authors:** Zaida Lentini, Álfora González, Eddie Tabares, María E. Buitrago, Maria Wêdzony

**Affiliations:** ^1^Center of Specialized Natural and Biotechnological Ingredients (CINEB), School of Natural Sciences, Universidad Icesi, Cali, Colombia; ^2^School of Natural Sciences, Universidad Icesi, Cali, Colombia; ^3^Institute of Biology, Pedagogical University of Cracow, Kraków, Poland

**Keywords:** gynogenesis, embryo formation *in vitro*, unpollinated ovule culture, doubled haploids, modern breeding methods

## Abstract

Cassava (*Manihot esculenta* Crantz) is an important crop for subsistence farming in tropical and subtropical regions. There is a need to increase the rate of genetic gain to develop varieties adapted to new environmental conditions affected by climate change, which also influences the patterns of pests and diseases. The rate of cassava genetic improvement is limited by the difficulty in obtaining true-breeding types (inbred/homozygous lines). Cassava inbreeding obtained through conventional sequential self-pollination increases exposure of useful recessive traits and breeding value of progenitors. However, it takes 10–15 years to produce homozygous lines through successive self-pollination. Doubled haploid (DH) technology is a functional alternative to progressive self-pollination, and is already widely used in major crops to accelerate inbreeding. This work aimed at developing a protocol for the culture of isolated ovules and the induction of gynogenesis in cassava. Basic groundbreaking studies on cassava embryo sac development are presented. A protocol using unpollinated ovules collected from ovaries 1 day after anthesis is described. In the unpollinated-cultured ovules, the presence of embryos formed probably from the egg cells and not surrounded by the endosperm, was documented by anatomical analyses. This achievement is an important first step in the development of a reproducible gynogenesis protocol for the generation of doubled haploids in cassava. This protocol can also be useful as a starting point to obtain DHs using alternative methods of induction such as pollination of cassava with pollen of distant species or with cassava pollen irradiated with gamma rays.

## Introduction

Cassava (*Manihot esculenta* Cranz) is the staple food for more than 750 million people living in tropical and subtropical regions. Its importance has been increasing rapidly due to fast population growth and the sharp increase in the use of this crop by the starch industry (Ceballos et al., 2004, 2012). Farmers have used vegetative multiplication for centuries. It preserved high levels of heterozygosity in hundreds of cultivated clones (Wang et al., 2011). Cassava breeding involving self-pollination or backcrossing had not been performed until recently (Ceballos et al., 2015). Interest in cassava flowering behavior and seed production was barely perceptible before 2000, but recently sparked increasing interest (Ceballos et al., 2017). The need for increased genetic enhancement and broadening the genetic base in cassava is well documented and justified (Ceballos et al., 2012, 2015, 2016). Successive self-pollinations are feasible, but since each cassava generative cycle lasts about 1.5 years, it would take from 10 to 15 years of work to produce S_5_ or S_6_ genotypes that could be used as progenitors in the production of hybrid seed (Ceballos et al., 2015). This is too long for current needs for cassava improvement by breeding. Doubled haploid (DH) technology is the best-known method to obtain genetically pure inbred lines in one step lasting from 1 to 2 years, depending on the method and the crop (Khush and Virmani, 1996; Germana, 2009; Wedzony et al., 2009; Murovec and Bohanec, 2012; Rudolf-Pilih et al., 2019). DH techniques are routinely applied to main staple food species (with the exception of bananas), including vegetatively propagated crops such as potato (Rokka, 2003; Seguí-Simarro, 2016), which encouraged us to develop an analogous technique for cassava.

The main step in DH technology covers the induction of sporophytic development in gametic cells to form homozygous plants. Among the key benefits of this approach is the ability to make full use of designed heterotic systems in breeding, to fix and fully express recessive traits and to use conventional backcrossing. As a breeding tool, DH technology could facilitate trait introgression and simplify systems for mutagenesis, transformation and gene editing, as well as support basic research, including biochemical, physiological, genomic, and phenomic studies (Ceballos et al., 2016). Since homozygous genotypes breed true, germplasm can be conserved as botanical seed. Facilitated exchange of germplasm could have a large impact through synergies among cassava breeding programs.

The best examples of a successful use of DH technology in breeding are reported from anther or microspore culture (i.e., plant androgenesis). However, reports indicate that gynogenesis may also be an efficient approach, especially when the androgenic response is not satisfactory (Kantartzi and Roupakias, 2009). Initial work on DH development for cassava started with androgenesis (Wang et al., 2011; Perera et al., 2014a, b). Despite success inducing multi-cellular homozygotic structures from anther and microspore culture (Perera et al., 2014a, b), further progress has not been reported.

Several methods to obtain haploid plants from the female gametophyte, which can be considered as an alternative to androgenesis, were revised by Germana (2009) and Wedzony et al. (2009). Among them, gynogenesis *in vitro* seems to be an attractive option, since it was successfully applied in several cultivated plants for which androgenic methods were not feasible (San and Gelebart, 1986; Keller and Korzun, 1996; Mukhambetzhanov, 1997; Bohanec, 2009). Efficient gynogenesis protocols generating a large number of embryos from female gametic cells can be obtained by culturing unpollinated ovaries/ovules or complete flowers (Olesen et al., 1988; Campion and Alloni, 1990; Ferrant and Bouharmont, 1994; Bohanec et al., 1995; Bohanec and Jakse, 1999; Michalik et al., 2000; Jakse and Bohanec, 2003; Alan et al., 2004). Haploid plants of various monocots (for instance, onion) and dicots (for example, beets, lemon, cotton) were obtained via flower or ovule cultures *in vitro* (Kantartzi and Roupakias, 2009; Wedzony et al., 2009). So far, the only way to obtain DH in sugar beet and onion is through gynogenesis (Bohanec, 2009), and gynogenesis seems to be the most relevant for cassava from a practical point of view. Sugar beet (*Beta vulgaris* L.) DH can be obtained by ovule culture, either directly by parthenogenesis when the egg cell develops into embryo and plant (Olesen et al., 1988; Ferrant and Bouharmont, 1994) or via plant regeneration from haploid calli proliferating from induced haploid embryos (Bossoutrot and Hosemans, 1985). Flower-bud *in vitro* culture in various cultivars of onion (*Allium cepa*) is another well-studied case of gynogenesis (Campion and Alloni, 1990; Bohanec et al., 1995; Bohanec and Jakse, 1999; Michalik et al., 2000; Jakse and Bohanec, 2003; Alan et al., 2004). Studies in onion indicate that the egg cell is the predominant source of haploid embryo in this species (Musiał et al., 2001, 2005).

There is a general assumption that, in contrast to *in vitro* androgenesis, it is possible to induce a gynogenic response from ovules over a broad range of developmental stages (Bohanec, 2009; Chen et al., 2011). It has been reported in several species from various genera (onion, sugar beet, squash, sunflower, gerbera, *Hyoscyamus muticus*, and *Melandrium album*) that immature embryo sacs have a higher gynogenic response (Bohanec, 2009). Nonetheless, the majority of the studies do not include analysis documenting the stages of embryo sac development suitable for gynogenesis. The best-documented case is the study in onion (Musiał et al., 2001), which suggested that young 2–4 nucleate embryo sacs were more responsive than mature stages. However, it was later demonstrated that these early developmental stages of onion embryo sacs mature after culture *in vitro* and that gynogenesis appears to be triggered once the embryo sac is mature (Musiał et al., 2005). It has also been found that sugar beet and sunflower embryo sacs are well organized and mature several days before the flowers open (Yang and Zhou, 1982), which enables gynogenesis induction before anthesis. On the other hand, Van Geyt et al. (1987) reported a degeneration of premature sugar beet ovules after a few days in culture.

Various factors had been reported to trigger gynogenesis (Bohanec, 2009; Chen et al., 2011). No apparent stress treatments have been used in the majority of protocols, although in some cases cold (sugar beet, Gurel et al., 2000) or heat treatment (cucumber, Gémes-Juhász et al., 2002) promoted gynogenesis. Bohanec (2009) noticed that the media used for gynogenesis induction are those used for micropropagation, since they are better designed for supporting the growth requirements of haploid embryos rather than for re-programming the gametophytic pathway into a sporophytic development, which is required for androgenesis. Plant growth regulators and synthetic analogs are postulated to trigger gynogenic embryogenesis. However, their quantity and composition vary considerably among known protocols and species. Therefore, for this study in cassava, it was necessary to experiment with various compounds.

This work describes a protocol for the culture of isolated ovules and the induction of gynogenesis in cassava. We aimed at an *in vitro* induction system triggering unpollinated female gametic cells to develop into embryos and subsequently into plants. Information on embryo sac maturity at anthesis was required to develop the *in vitro* gynogenesis protocol. In contrast to other staple crops, studies of cassava female flower development were limited, which caused problems when beginning the present study. Therefore, the stage of cassava embryo sac development in relation to bract opening (considered as the anthesis day for this species) and the various stages of ovule response to culture conditions were determined. Here, we report the results from our studies using unpollinated ovules collected 1 day after anthesis (DAA), selected as the most promising to react to the induction culture.

## Materials and Methods

### Plant Material and Growth Conditions

For the purpose of the present study, HMC-1 and CM 7951-5 were selected from a group of cassava clones of economic importance in Colombia. Their flower production is profuse in relation to other clones, and their flowering cycle already starts 5–6 months after planting. These clones were obtained from the CIAT (International Center for Tropical Agriculture, Cali, Colombia) Cassava Breeding Program.

Well-established nurseries were grown at the Palmira experimental station of CIAT in fertile soils, with adequate rates of macro- and micro-elements for cassava growth and development, under controlled phytosanitary conditions, with minimum or no chemical applications for insect, disease, and weed control. The average temperatures of 18°C at night and 27°C during the day were recorded. Plants were rainfed and irrigated when required.

**FIGURE 1:**
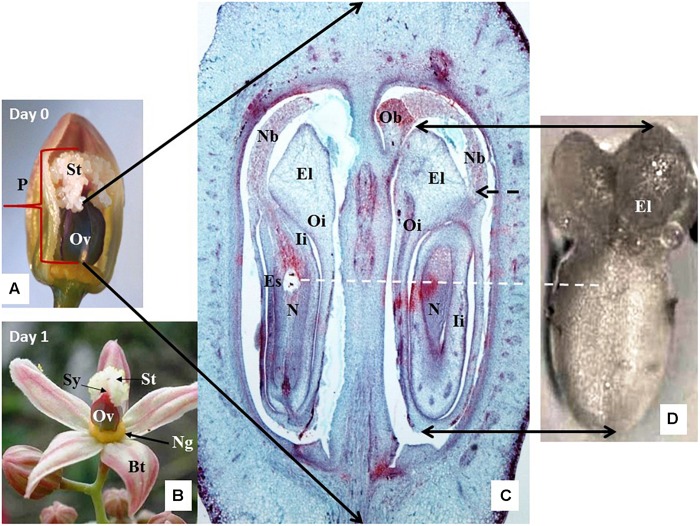
Anatomy and histology of cassava female cyathium. **(A)** A cyathium with two out of five bracts removed to expose its pistil (P) with large ovary (Ov) and feathery white stigma (St). Material collected just before bract opening, on the day considered as the day of anthesis (Day 0). **(B)** Cyathium collected the next day after the bracts (Bt) opened, i.e., the first day after anthesis (1 DAA). The female cyathium of cassava is reduced to a single pistil (P) and circle of yellow nectar glands (Ng) just below the ovary (Ov). The short style (Sy) neck is hardly visible under the white feathery stigma forming three lobes. **(C)** A cross-longitudinal section of the ovary as indicated by the black arrows. Two out of three ovules are visible, sectioned at different angles. One ovule is present per loculus. Outer integuments (Oi) form large elaiosomes (El), also called caruncles, are visible as well as in (D). Inner integuments (Ii) form a thick layer around the nucellus (N). The nucellus protrudes through the micropyle with a long nucellar beak (Nb), whose end tightly touches an obturator (Ob) (more visible on the top end of the right loculus). The position of the embryo sac (Es) is visible in the ovule of the left loculus. **(D)** A dissected ovule, isolated for culture and observed from the abaxial side. The adaxial side of the ovule is placed on the solid-culture medium. Black-dashed arrow shows the site where the beak, now broken, should appear. White-dashed line marks the most probable position of the embryo sac in the dissected ovule.

According to breeders’ reports (Hernán Ceballos, personal communication, CIAT), cassava increases fertility from the second flowering event and beyond. Cassava inflorescences are cyathia (singular cyathium), one of the specialized pseudanthia (“false flowers”) forming the inflorescence of plants in the genus *Euphorbia* (*Euphorbiaceae*). In cassava, cyathium structures are reduced to a single style wrapped in five petal-like bracts (Figures 1A,B). Female cyathia from the third or fourth flowering event of healthy-looking and vigorous plants, with profuse cyathia formation of similar morphology and developmental stage, were used for the experiments. Tightly closed cyathia were marked and covered with mesh bags 1 day before opening (anthesis) or early in the morning (0700–0900) on the anthesis day at the latest to avoid accidental pollination. Bract opening usually occurs naturally around noon or in early afternoon. The first day that the bracts open naturally will be referred to herein as the day of anthesis or Day 0. The isolating bags remain on the cyathia until sample collection. Thus, the protocol prevents the undesirable pollination of female flowers collected for *in vitro* culture.

### Determination of Embryo Sac Developmental Stages

Cyathia were collected on the day of anthesis (Day 0) and 1 or 2 DAA to determine the stage of development of the embryo sacs to be used for the ovule cultures. The cyathia were always collected at 1300–1400 and placed in zip-plug bags within a Styrofoam cooler with refrigerant gel, and then they were transported from the field to the laboratory within the first hour after collection.

About 30 pistils (10 per day studied, Day 0, 1 DAA, and 2 DAA) were sectioned, providing about 30 ovules for anatomical analyses using histological sections per collection time. The samples were kept cool for 1–2 h until fixed in FAA [formaldehyde (Sigma-Aldrich F8775): glacial acetic acid: (Sigma-Aldrich 537020): absolute ethanol solution 1: 3: 6 v/v/v)] for at least 24 h. Then, the samples were submerged for a minimum of 24 h in 70% ethanol to eliminate most of the formaldehyde and acetic acid. The samples could be preserved in 70% ethanol for several weeks before further processing. Before paraffin embedding, the samples were dehydrated in a gradient of absolute ethanol: tertiary butanol (Sigma-Aldrich471712) in seven steps of 1.5 h each, ending with pure tertiary butanol, and then they were embedded in paraffin blocks according to Ruzin (1999). Longitudinal 4-μm- or 8–10-μm-thick sections (depending on the age of the ovaries) were made with an American Optics Spencer 820 Rotary Microtome. The sections were placed on microscopic slides, dried at 60°C for at least 1 h, and then stained with Safranin O-Fast Green (Fernandez-Luqueno et al., 2008). The samples were analyzed under light and dark field illumination with a Nikon Eclipse 55i microscope. Photographs were taken with a high-resolution Nikon DS-Fi1 5-megapixel cooled color digital microscope camera, coupled with a NIS-Element imaging for acquisition, analysis, and visualization of the microscopy data.

### Ovule Isolation and Culture

Based on the results from the study on the stage of development of embryo sacs, the unpollinated cyathia for *in vitro* culture were collected in the field on Day 0 or 1 DAA. The cyathia were surface-sterilized with 70% ethanol for 1 min, followed by 1.5% sodium hypochlorite solution in water, with 3 drops of Tween 20 for 17 min, and rinsed four times with sterile distilled water. In the sterile environment, the cyathia were further dissected under a stereomicroscope (Nikon C-LEDS and cold-light Nikon NI-150).

Three culture procedures were evaluated aiming at inducing gynogenic embryo formation and avoiding injury of the isolated ovules and callus formation from the walls of the cultured ovules (i.e., integuments and nucellar tissue).

(A) Isolated ovule culture: the pistils were cut off the cyathia just above the nectar glands (Figure 1B). Then, ovules (3 per ovary) were extracted gently from the loculi with a minimum of physical damage by a series of longitudinal and transversal cuts of the ovarian wall. Isolated ovules (Figure 1D) were cultured either in the liquid medium or with the adaxial side downward the solid medium.

(B) Ovary culture: pistils were cut off the cyathia just above the nectar glands as in (A) and then cultured either with or without stigma. In the latter case, the stigmas (Figure 1A) were cut off from the ovary at the level of the style neck. In both variants, the ovaries were placed with the basal cut end on the medium.

(C) Carpel culture: excised ovaries without nectar glands and without stigmas were cut longitudinally along the carpel walls in three sections containing one ovule each in their loculus. The carpels were cultured with the basal cut end on solid medium.

In protocols (B) and (C), once the ovules protruded through the carpel walls (usually about 3–4 weeks after the start of the culture), they were isolated and placed on fresh medium of the same composition as in (A), with the adaxial side down on the medium.

### Culture Media

Medium composition for the gynogenesis induction was tested in a single-step induction system or in a sequence (two-step induction system). For the single-step induction system, cultures using methods A, B, or C (described above) were plated on liquid or solid (solidified with 4% Gelrite^TM^ ) media. Four media compositions, CBM (Gémes-Juhász et al., 2002), F6 and A1 (Michalik et al., 2000), and a modified BDS (Bohanec and Jakse, 1999) previously reported for cucumber and onion gynogenesis, were evaluated (Supplementary Table S1). All the media were supplemented with 2 mg/L 2,4-D, 2 mg/L BAP, and 10% sucrose (pH 5.8). The cultures were kept on each medium for 3 weeks and then sub-cultured on the same but fresh medium for another 3 weeks. The cultures were kept at 28–30°C in the dark.

For the two-step induction system, carpel cultures (method C) were plated on solid F6 or BDS medium for 3 weeks. The ovules were then isolated from the carpels (Supplementary Table S1) and cultured on fresh F6 or BDS medium, respectively, for another 3 weeks. Afterward, the ovules were transferred to R medium (Michalik et al., 2000) containing 1 mg/L NAA, 2 mg/L 2ip, 200 mg/L proline, and 10% sucrose for a total of 12 weeks (Supplementary Table S1). Subsequently, the ovules were sub-cultured on MS salts and organics without growth regulators, supplemented with 10% sucrose and 0.8 mg/L CuSO_4_ × 5H_2_O, which is a 32-fold increase in Cu with respect to the original MS medium (Murashige and Skoog, 1962), for at least 6 weeks. Increased amounts of Cu were reported to increase embryogenesis and shoot and root growth of cassava tissue culture *in vitro* (Schöpke et al., 1992; Danso and Ford-Lloyd, 2002). The cultures were kept at 28–30°C and in the dark when cultured on solid F6 or BDS medium, and then in a 12 h day/night photoperiod with light energy of 80–100 μmol.m^–2^.s^–1^ during the day when cultured on R medium.

Twelve ovules were plated in 9-cm-diameter petri dishes. The petri dishes were sealed with a food seal wrap-paper (a clinging food foil) to prevent drying. Three hundred and sixty ovules were set up for each of the treatment combinations per experiment. The experiments were triplicated.

The cultures were evaluated by assessing changes in the ovule sizes, colors, occurrence of callus formation, and structures appearing from the surface or from the inside of the cultured ovules, including necrosis of integuments and other tissues. The cultures were evaluated using different parameters: the frequency of callus induction (number of ovules with calli/100 ovules), the degree of ovule necrosis (number of ovules with signs of necrosis/100 ovules), and callus necrosis (number of calli per ovule with necrosis/100 ovules). In addition, the degree of callus induction measured by the visual estimation of the relative ovule surface area covered by calli (0, 25, 50, 75, or 100% of the ovule surface/100 ovules) was also considered.

Thirty ovules per treatment selected at random were used for histology analyses by free-hand sectioning and 25 ovules for sectioning in paraffin. The latter were prepared in the same way as described above for the studies to determine the stage of development of embryo sacs. Free-hand sections were prepared with thin scalpels and analyzed under a high-resolution Stereo Microscope (SMZ800). The paraffin sections were analyzed under light and dark field illumination with a Nikon Eclipse 55i microscope. Photographs were taken with a high-resolution Nikon DS-Fi1 5-megapixel cooled color digital microscope camera, coupled with a NIS-Element imaging for acquisition, analysis, and visualization of the microscopy data.

### Data Analysis

Analysis of variance (ANOVA) was performed for callus induction and ovule and callus necrosis. In all cases, the analyses were performed using the SAS statistical program, software version 9.4. The means were compared by Tukey’s Studentized Range (HSD) test. To maximize the power of the analysis, all treatments were compared with each other at a confidence level of 0.05. In the figure reporting the results of these analyses, different letters were assigned to the different treatments showing a significant difference at a *P*-value = 0.05 according to the contrast output.

## Results

### Embryo Sac Stage of Development

A distinct pistil with feathery-like stigma characterizes the cassava female cyathium. This structure is clearly visible on Day 0 prior to bud opening (Figure 1A). The style neck is very short (Figure 1B) in relation to the large ovary with thick walls (Figure 1C). The ovary is trilocular, and each loculus contains one ovule (Figure 1C). Cassava has two specific anatomical features: an outgrowth of the ovary central columella called the obturator (Ob) and the very long outgrowth of the nucellus called the nucellar beak (Nb) that protrudes from between the integuments and reaches the Ob (Figure 1C). The outer and inner integuments are thick and composed of many cell layers. The thick endings of the outer integument are called elaiosomes or caruncles (Figures 1C,D), and they are characteristic for numerous *Euphorbiaceae*. The elaiosomes are also clearly visible on isolated ovules prior to culture (Figure 1D).

**FIGURE 2:**
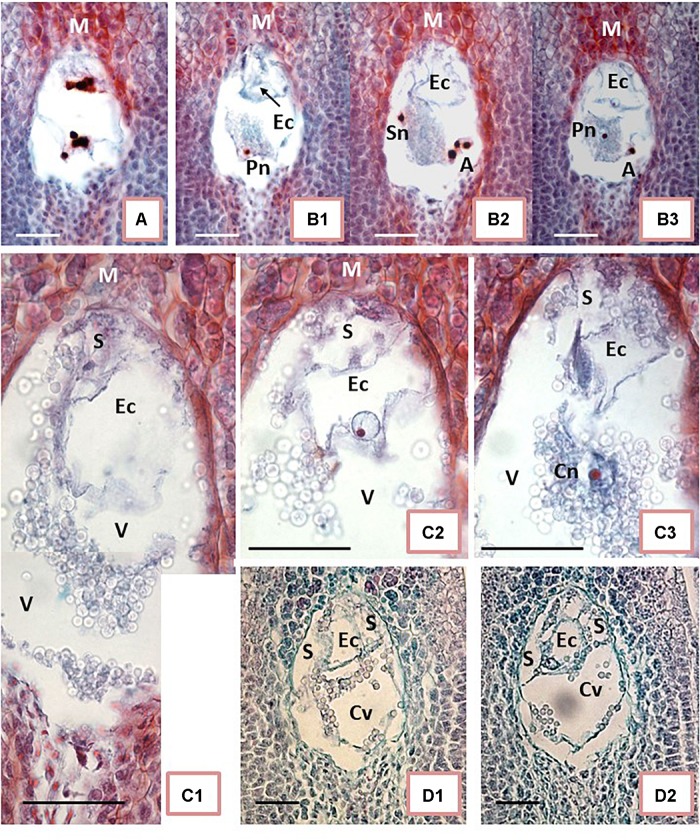
Embryo sac development of unpollinated ovules. **(A,B)** On the day of anthesis (Day 0, the day of the first opening of bracts). **(C)** One day after anthesis (1 DAA). **(D)** 2 DAA. **(A)** Shows 8-nucleate embryo sac (ES). At the micropylar (M) end (top end of ES in the picture), the nuclei are in late anaphase-telophase stage. The nuclei at the chalazal end of ES already completed divisions. **(B1–B3)** Show ES during cellular organization. Synergids are not yet formed. One of the potential synergid nucleus is visible on **(B2)** close to the left ES wall. An outline of the egg cell (Ec) is visible at the M end on **(B1–B3)**. Outline of the Ec is visible in each picture, whereas the Ec nucleus is visible on **(B3)** exclusively. **(B2)** Shows a group of three nuclei in the lower right corner (putative antipodals), and **(B3)** shows a cut-off fragment of one nucleus at the corresponding position. Two polar nuclei are visible separately and at a distance: the first one in the bottom-left corner of ES **(B1)** near a central starch clump, and the second one in the middle of starch grains, close to the ES center **(B3)**. **(C1–C3)** Correspond to three sections in sequence showing fragments of ES. The highly vacuolated Ec is visible at the M end. The basal end of Ec is attached eccentrically to the ES wall, slightly to the right of its top **(C1–C3)**. The Ec nucleus is visible only in **(C2)**. A fragment of the synergid is visible above Ec to the left. The second synergid is not visible on the presented sections. Either a polar nucleus or the polar nuclei already fused into the secondary nucleus of the central cell of ES are visible in C3. **(D1,D2)** show further EC apparatus organization; two synergids are visible on both sides of Ec. **(A,B,D)** present photos taken with 40x objective while **(C)** had an objective of 100x. Bar = 20 μm. (Ec) egg cell; (S) synergid cell; (Sn) synergid nucleus; (A) antipodal cells; (Pn) polar cell nucleus; (Cn) central cell nucleus; (V) vacuole; (Cv) central cell vacuole.

Cassava embryo sacs (ES) are large. Their content was visible on 5–9 subsequent sections 8 μm thick on Day 0 (Figure 1C) or from 7 to 12 sections 9 μm thick on 1 DAA and 2 DAA. Therefore, the key ES elements, such as the egg apparatus, had to be analyzed on the several sequential sections. The selected sections presented in Figure 2 illustrate some key elements of ES during development on Day 0, 1 DAA, and 2 DAA.

The ES showed an oval shape on the longitudinal sections. In samples fixed on Day 0 (Figures 2A,B), the ES were of variable sizes. The smallest diameters were 40 μm × 60 μm in width × length while the largest diameters were 75 μm × 108 μm. ES of various sizes could be found in sections of the same ovary, within ovules not differing noticeably in size and shape. Therefore, we concluded that ES development of different ovules from the same ovary on Day 0 was not synchronous. Moreover, on Day 0 (day of anthesis), ES developmental stages varied from two-nucleate ES or early stages of cellular organization (Figure 2A) up to almost mature ES (Figure 2B). To some extent, the ES sizes might be correlated with their developmental stage. It appears that the smaller the ES, the earlier its developmental stage. However, a larger number of samples needs to be analyzed in order to confirm this hypothesis statistically (Figures 2A,B). The nuclear divisions in the four-nucleate ES were not always synchronous (Figure 2A). At this stage, nuclear fusion was not found and all nuclei in the ES were of similar sizes, but distinctly larger than the nuclei of the surrounding nucellar tissue. ES vacuolization was profound already at the four-eight nucleate stage (Figure 2A). At the onset of ES cellularization and formation of the typical three-cellular egg apparatus, antipodals were not clearly visible (Figure 2B).

Starch accumulation in the cytoplasm of the central cell was the most noticeable change in ES on 1 DAA (Figure 2C). It was difficult to examine ES cells and nuclei due to the dense cytoplasm and the starch grain clumps increasingly filling the central cell. ES enlarged further to the widest diameter, reaching 130 μm, and the longest length, 200 μm. The key elements of the egg apparatus were found in most ES on 1 DAA. Thin walls and the large size of the egg apparatus cells, however, made detailed analysis difficult (Figure 2C). As maturity progressed from 1 DAA (Figure 2C) to 2 DAA (Figure 2D), the cell walls became more visible and starch grains accumulated, surrounding the egg apparatus. The egg cell was vacuolated and larger than the synergids. The egg cell nucleus was usually located adjacent to the thin cell wall separating it from the central cell (Figures 2C2,D2). The polar nuclei were usually fused (Figure 2C3) and surrounded with dense clumps of starch granules. Antipodals, if present at all at this stage, were hardly visible.

### Effect of the Culture Method on Gynogenesis Induction

**FIGURE 3:**
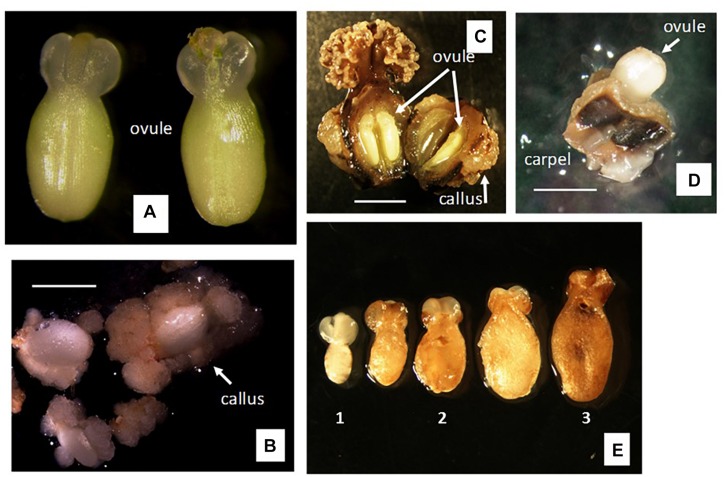
Responses to gynogenesis induction of unpollinated female organs using different methods of culture. **(A,B)** Method A of culture, i.e., isolated ovule culture using single-step induction system. **(A)** Ovule dissected 1 day after anthesis (1 DAA). **(B)** Response after 4 weeks of culture on solid medium of ovules isolated as in **(A)**. **(C)** Method B of culture, i.e., ovary culture using single-step induction system. Left, pistil culture after cutting off the nectar gland ring. Right, ovary culture after cutting off the nectar gland ring and the stigma. Cultures spontaneously opened the ovary wall after 3–4 weeks of culture on solid medium, exposing ovules within ovary. **(D,E)** Method C of culture, i.e., carpel culture. Individual carpels cut off from the ovary and cultured containing one ovule each. **(D)** Ovule protruded out of the carpel wall after 3–4 weeks of culture on solid medium. **(E)** (1) Freshly dissected ovule from the cultured carpels as shown in **(D)**; (2) ovule after 6 weeks of culture on solid F6 or BDS medium, using single-step induction system; (3) ovule after 6 weeks of culture on solid F6 or BDS medium, followed by 9 weeks on R medium, using two-step induction system. Bar = 5 mm.

After 4 weeks of culture, profuse callus formation was noted when isolated ovules were cultured right after dissection from the pistil using method A (Figures 3A,B). Isolated ovules showed increased sizes and most of them were covered by friable calli not only at the places where they were wounded during the excision process from the ovary but also around the outer integument (Figure 3B). In some cases, calli protruded from the nucellar tissue inside the ovule. Ovary culture (method B), with or without stigma attached, showed profuse callus formation on the ovary wall after 3 weeks of culture (Figure 3C). Ovules cultured in ovaries with stigma were of similar sizes as on the first day of culture, without any noticeable morphological or anatomical changes (Figure 3C, Left). On the other hand, ovules developed further and enlarged when cultured in ovaries without stigma (Figure 3C, Right).

In contrast to the two previous methods of culture, no callus formation was noticed on either the carpel walls or the ovule surface (outer integument surface) when using method C (i.e., carpel culture). Using this method, the ovules increased significantly in size and spontaneously protruded from the carpels about 3 weeks after culture started (Figure 3D). Once the ovules protruded from the remnants of the carpel wall, they were excised and transferred onto fresh culture medium. However, most ovules did not progress further in development, maintained the same size and color as seen on the day of dissection from the carpel wall (Figure 3E1), and eventually collapsed and degenerated. This took place except for some cultures in which the ovules continued growing (Figure 3E2) and showed external morphological and color changes. In most cases, the outer integuments of these ovules turned brown and brittle, exposing the inner integument (Figure 3E3).

### Effect of Culture Medium on Gynogenesis Induction

**FIGURE 4:**
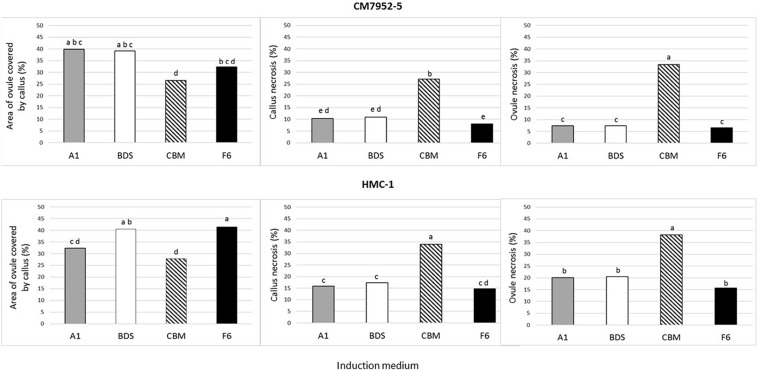
Effect of culture medium on gynogenesis response of unpollinated isolated ovules after 4 weeks of culture using Method A and single-step induction system. The effect was measured as area of ovules covered by calli, callus necrosis, and ovule necrosis. To maximize the power of the analysis, mean values were calculated for the pooled data of 360 ovules cultured on each solid and liquid medium, for 720 data points per medium composition. The means were compared by Tukey’s Studentized Range (HSD) test. Values with identical letters indicate no significant difference at a *P-*value = 0.05.

Four weeks from the start of culture, all isolated ovules (method A of culture) of both tested clones showed profound callus formation independently of the medium composition and state (liquid or solid). However, differences occurred in the degree of callus induction measured by the relative ovule area covered by calli (0, 25, 50, 75, or 100% of the ovule). Liquid medium induced significantly (*P* = 0.05) more callus proliferation (i.e., about a 1.6-fold increase) (average 42.6% ± 0.7) in comparison with the effect seen on solid medium (27.3% ± 0.4). Likewise, CBM medium induced the least (*P* = 0.05) proliferation of calli (27.3% ± 0.5) in relation to A1 (36.1% ± 0.9), F6 (36.8% ± 0.9), and BDS (39.8% ± 0.9) media. Nevertheless, CBM medium induced the highest necrosis of both ovules and calli in both genotypes. In general, this pattern is seen when comparing the responses with each genotype in all media (Figure 4). HMC-1 showed a higher level of ovule and callus necrosis (23.7% ± 0.9 and 20.6% ± 0.8, respectively) than CM 7951-5 (13.6% ± 0.7 and 14.1% ± 0.7, respectively). CM 7951-5 showed the least necrosis on F6 medium, followed by BDS and A1 media (Figure 4).

### Embryo Formation in Unpollinated Ovules Using Carpel Culture

Carpel cultures of CM 7951-5 unpollinated ovules were tested at two different stages of development of the embryo sac. Ovules harvested on Day 0 did not respond at all and usually degenerated several weeks after culturing. Ovules from cultures started on 1 DAA showed changes in morphology and gynogenesis induction (embryo formation) and this was documented with histological analyses.

**FIGURE 5:**
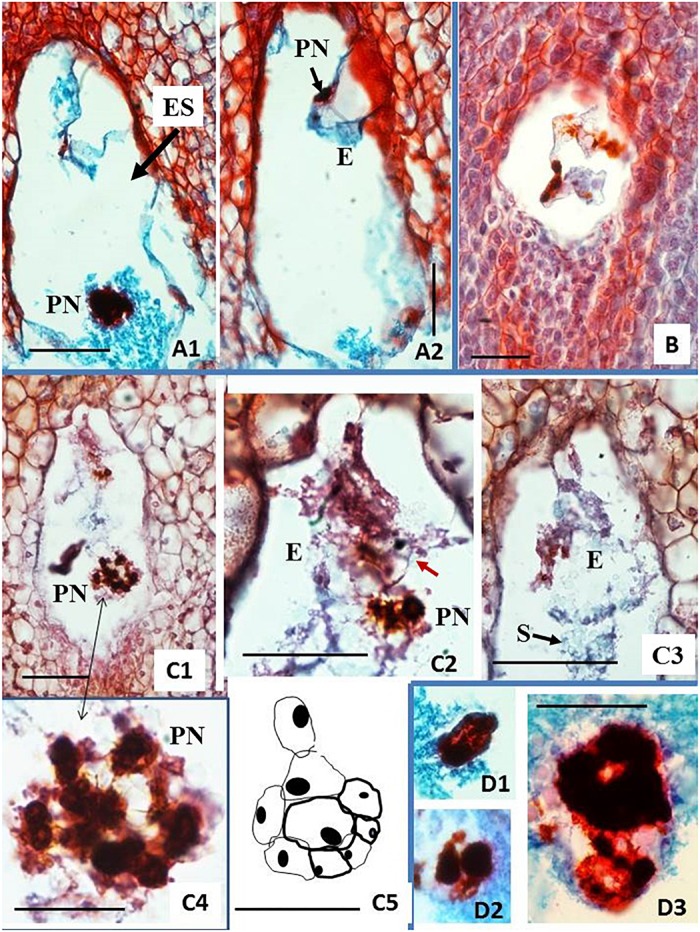
Histological analysis of fixed, embedded, and sectioned unpollinated ovules from carpel cultures using Method C and single-step induction system. Sections of ovules cultured on solid BDS medium **(A,B)** or on solid F6 medium **(C,D)** for 6 weeks. **(A1,A2)** Embryo sac (ES) showing two-cell proembryo (E) at the micropylar (M) end and highly polyploid nucleus (PN) surrounded by dense cytoplasm at the chalazal end of ES. **(B)** Relatively small ES with three strongly stained nuclei lying in line and accompanied by cytoplasm containing starch grains. **(C1–C5)** ES containing a globular embryo and large groups of chromatic structures, probably polyploid nuclei. Epidermal layer of cells of the embryo is already differentiated. Red arrow in **(C2)** points at the embryo-epidermal layer of cells that can be seen of this section. The scheme drawn in **(C5)** is a representation of the whole embryo shown in **(C2)**. The embryo reconstruction was done by drawing the embryo cells walls, overlapping the images of the sequential sections containing this globular embryo. Dense clumps of starch (S) granules are clearly seen in **(C3)**, covering the globular embryo. Large chromatin structures are located at various positions of ES. Enlargement of the group of nuclei from **(C1)** shown in **(C4)** (doubled pointed black arrow) made chromatin bridges visible. **(D1–D3)** Show enlarged chromatin structures similar to those in **(C1–C4)** found in various other ES. Paraffin sections 8 μm thick, stained with the Safranin O-Fast Green method. The bar equals 1 mm on **(A1,A2,B,C1)**, and 0.5 mm on **(C2–5,D)**.

One of the 25 sectioned ovules for histological analyses of clone CM 7951-5 cultured on solid BDS medium for 6 weeks showed formation of a two-cell embryo (Figures 5A1,A2 and Table 1). And, one of the 25 sectioned ovules for histological analyses of clone CM 7951-5 cultured on solid F6 medium for 6 weeks showed formation of a globular embryo (Figures 5C1–C5 and Table 1). Both embryos were formed at the micropylar pole of the respective embryo sacs. In both cases, the embryos were accompanied by various numbers of strongly stained nuclei of various sizes located in the thin layer of cytoplasm close to the embryos (Figures 5A2,C1,C2,C4). Similar nuclei were also found in some other analyzed ES without detected embryos (Figure 5B). The nuclei stained strongly, and they were often grouped close to each other. The enlarged image allowed us to observe thin chromatin links (chromatin bridges) between nuclei (Figure 5C4). Some of these nuclei were vastly enlarged, and probably highly polyploid (Figures 5A1,C1,D1–D3). The structure of chromatin in those enlarged nuclei pointed to nuclear fusions or disturbed mitoses as the mechanism of polyploidization. No embryo formation was found in any of the sections analyzed from CM 7951-5 cultured on CBM or A1 medium, and in any of the HMC-1 samples (Table 1).

**TABLE 1:** Responses of unpollinated ovules cultured in carpels (Method C) to conditions aiming at induction of embryo formation (gynogenesis *in vitro*).

Method of histological analyses	Culture media^a^	Culture length (weeks)	Studied ovules	Embryos found, total (%)	Stage of embryo development
Paraffin sections	BDS^b^	6	25	1 (4%)	2-cell proembryo
	F6^b^	6	25	1 (4%)	1 globular
Free-hand sections	BDS/R^c^	6/9	30	5 (16.7%)	5 globular
	F6/R^c^	6/9	30	8 (23.7%)	7 globular, 1 torpedo

*^a^Solid medium. ^b^One-step induction system. Ovules were cultured on BDS or F6 medium for 6 weeks. ^c^Two-step induction system. Ovules were initially cultured on BDS or F6 medium for 6 weeks and then transferred to R medium for 9 weeks.*

**FIGURE 6:**
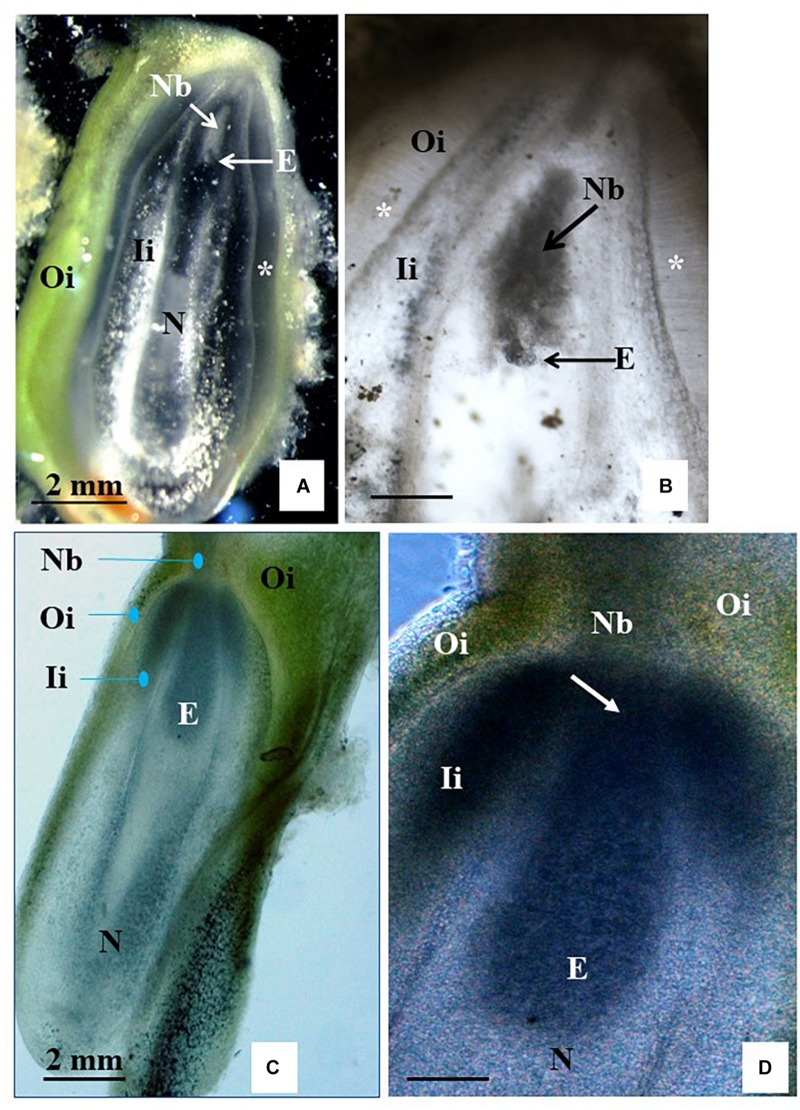
Free-hand longitudinal sections of unpollinated ovules from carpel cultures using Method C and two-step induction system. Sections of ovules cultured on solid BDS medium **(A**,**B)** or on solid F6 medium **(C**,**D)** for 6 weeks and then transferred to R medium. **(B)** Shows an enlargement of the micropylar side of the section **(A)** to expose the structure better, which resembles a globular embryo (E, pointed to by arrow). The outer integument (Oi) shows chlorophyll, while its fragment lying on the medium started callusing. The inner integument (Ii) is mostly degenerated without traces of callus formation. Cell wall fragments glitter in the dark field illumination. As during natural process of testa formation, cells of inner epidermis of Oi elongated perpendicularly to the surface, forming a distinguished layer (marked with small stars). Nucellus (N) and especially nucellar beak (Nb) accumulated starch grains. Embryo sac is visible as an empty cavity (black in **A** and translucent white in **B**). **(C)** Shows a slight spot of callus on the Oi. The Oi remained green similar to **(A)**. **(C,D)** Show a torpedo-stage embryo that is closely attached to Nb. **(D)** An arrow points to the site of the embryo attached to the starch-reach beak region at the micropylar side end of the embryo sac. Sections were obtained using freshly collected material and show their natural color. **(A)** Images in dark field illumination using an inverted Nikon ECLIPSE Ti-S microscope. **(B–D)** Images in light illumination using a Nikon Eclipse 55i microscope. Photographs were taken by placing the sections in drops of water covered with a cover slip.

Based on these results, clone CM 7951-5 and BDS and F6 media were selected for the next experiments using the two-step induction system. Carpel cultures were performed on F6 or BDS medium for 3 weeks. Then, the ovules were isolated from the carpel and sub-cultured on fresh F6 or BDS medium for another 3 weeks to complete 6 weeks of culture on the respective medium, and then transferred to R medium for another 9 weeks (15 weeks of culture in total). Histological analyses using free-hand sections of the non-fixed material indicated that 5 of the 30 ovules analyzed from BDS medium showed the development of structures resembling the globular stage (Table 1 and Figures 6A,B). Cultured ovules preserved chloroplasts in the outer integuments and accumulated starch in the inner integuments and the remnants of nucellar tissue, which indicates that they might support development of a gynogenetic embryo on these media, if any is formed (Figure 6). Moreover, 8 out of the 30 ovules analyzed from F6 medium showed embryo formation (Table 1). Seven of them showed structures resembling globular-shaped embryos and one showed a structure resembling a torpedo-shaped embryo at the micropylar embryo-sac pole (Figures 6C,D). No traces of developing endosperm-like tissue were found in these cases. The ovules were significantly enlarged in comparison to their size on the day of their isolation from the carpel and preserved chloroplasts. Some of the cultured unpollinated ovules grew comparably to immature seed growth, and starch was continuously accumulated within them. Despite efforts to maintain embryos to develop further and to trigger their differentiation into plants by transferring them from R medium to MS medium for at least 6 weeks as described in the two-step procedure, plant regeneration or further embryo differentiation was not achieved. The ovules did not grow further and there was no other indication that those ovules sustained further embryo differentiation leading to the full recovery of plants from these experiments.

## Discussion

Cassava has not been the subject of studies on gynogenesis until now. This work presents basic groundbreaking documentation on cassava embryo sac development. A protocol is described using unpollinated ovules collected from ovaries 1 day after anthesis. The study suggests that at this stage the embryo sac, and especially the egg apparatus, is fully formed. The use of carpel culture, rather than the culture of isolated ovules or complete ovaries (with or without stigma attached), prevented callus proliferation from the ovule integuments and induced the formation of embryos. The induction of embryos growing in unpollinated ovules and the absence of endosperm were documented by anatomical analyses in sections from different experiments and treatments. This achievement is a first step in the development of a protocol for the generation of DH via gynogenesis in cassava. It may also be useful as a starting point to obtain DHs using alternative methods of induction such as pollination with pollen of distant species or with irradiated cassava pollen.

Well-documented studies in other species have shown that gynogenic response depends on several biotic and abiotic factors. The main factors include the developmental stage of the female gametophyte at the time of ovule/ovary culture and the genotype and growing conditions of donor plants, in combination with culture conditions and culture media composition (Chen et al., 2011; Murovec and Bohanec, 2012). Of all those factors, the stage of development of the ES appears to have the greatest influence on gynogenic response.

Several studies recognized that the developmental stage of the ES is one of the most important factors affecting gynogenic induction (Yang and Zhou, 1982, Yang and Zhou, 1990; Muren, 1989; Mukhambetzhanov, 1997; Bhojwani and Thomas, 2001). The optimal developmental stage of ES for high gynogenic response varies widely depending on the species. Reports from 21 angiosperms suggest that the recovery of gynogenic doubled haploids might be possible from various ES developmental stages as diverse as from the uninucleate ES up to the mature ES stage (Bohanec, 2009; Chen et al., 2011). However, it is important to point out that most of these studies did not include histological analyses documenting the actual ES stage of development at the time of ovule culture. Studies with well-documented histological data indicate that the mature ES is regarded as the optimal stage for gynogenic response (Musiał et al., 2005). One of the reasons why histological analyses are generally not included when developing gynogenesis protocols appears to be the difficulty and time-consuming work needed. In contrast, determining the stages of development of immature pollen when developing androgenesis methods is relatively simple in most cases.

An important finding in this study is that ovules from the same ovary may be at different developmental stages. At anthesis, ES varied from two-nucleate ES, or early stages of cellular organization, up to almost mature ES in some ovules. The asynchrony of the developmental stage of ovules within the same ovary may pose difficulties for standardizing a protocol for high gynogenic response in cassava. The histological analyses also indicated that, despite efforts to use homogeneous samples, many ovules of the same shape and size probably did not contain the ES in the same stage of development. This asynchrony limits the number of ovules capable of reacting in the same way to conditions suitable for gynogenic induction. The not-synchronous ES development in the same ovary could also explain the reduced recovery of seeds in cassava from hand-made pollination (Yan et al., 2014; Ferguson et al., 2019).

Another factor to consider important for cassava gynogenesis might be the developmental stage of the female cyathia regarding the position of the flowering event. In summer squash, the position of female flowers on the plant stem significantly affects embryo induction from ovule cultures (Dong et al., 2016). According to breeders, cassava increases fertility from the second flowering event and beyond. In this study, female cyathia from the third or fourth flowering events of healthy-looking and vigorous plants, with profuse cyathia formation of similar morphology and developmental stage, were used. However, detailed analysis of the ES stage of development according to the position of the female cyathia in the inflorescences was not considered.

The histological analyses conducted in this work indicated that the accumulation of starch granules increases in the embryo sac as it matures. Starch accumulates in the ES during the preparation of the egg cell apparatus for fertilization. Therefore, the availability of high levels of carbohydrates might be crucial for induction, as well as for the sustained development of the embryos in unfertilized cassava ovules cultured *in vitro*. In this work, it was possible to induce cell division up to the formation of globular- to torpedo-shaped embryos from unpollinated ovules cultured *in vitro* on 1 DAA. Our work suggests that 1-DAA ovules contain the three-cell, fully formed egg apparatus. These results coincide with well-documented studies suggesting that the mature ES is optimal for gynogenesis induction (Musiał et al., 2005). Apparently, in species in which young stages of ES development have been proved more suitable, gynogenetic development usually starts only after the ES matures during culture (Bhojwani and Thomas, 2001; Musiał et al., 2005).

In this work, embryos were formed at the micropylar end of the embryo sacs. In other well-studied species, such as onion (Musiał et al., 2005), micropylar localization of the embryo has been interpreted as origination from the egg cell (induced parthenogenesis) or synergid (synergid apogamy). The cassava embryos documented in this work were found accompanied by some free nuclei located in a thin layer of cytoplasm close to the embryo, resembling the induction of endosperm development. Such numerous free nuclei were also found in some ES without a formed embryo. These nuclei stained strongly, were often grouped close to each other, were vastly enlarged, and were probably highly polyploid. Enlarged images allowed observing thin chromatin links between nuclei (chromatin bridges). The data generated do not yet allow a clear interpretation of this hypothetical polyploidization or the origin of nuclei themselves. It could be assumed that, in the reaction to the culture conditions, unfused polar nuclei or fused nuclei of the central cell of the embryo sac first undergo several mitotic divisions before the latter become disturbed. Thus, arrested mitoses or other mitotic aberrations are proposed as the mechanism of polyploidization. The structure of chromatin in the super numerical and enlarged nuclei also points to nuclear fusions or disturbed mitoses as the mechanism of polyploidization. In order to formulate a concluding proposal, ES development in culture should be further analyzed. Since the divisions are undergone without setting walls, the central cell is unlikely to provide an embryo-like structure or callus.

In this investigation, there was no sign of autonomous endosperm induction in ovules with or without embryo formation, as has been reported in other species, unless we do not consider aberrant nuclei in the central cell as the endosperm. Apparently, the development of an autonomous endosperm does not seem to be essential for the growth of gynogenic embryos. Autonomous endosperm and gynogenic embryo development often occurred in different ovules (Kashin et al., 2000; Musiał et al., 2001), except for millet and onion, in which autonomous endosperm and gynogenic embryos have been observed in the same embryo sacs. Likewise, the development of autonomous endosperm and embryo frequently occurs in the same ovule in systems in which irradiated pollen is used for haploid induction (Musiał et al., 2001). In this work, the cassava embryos induced from unpollinated ovules failed to develop beyond the globular or torpedo stages. Therefore, the need for autonomous endosperm development, as a crucial factor for the conversion of induced cassava embryos into plants, is yet to be examined. Other possible factors to be studied that potentially affect further embryo growth and differentiation are media composition, carbohydrate level, and growth regulator level and proportion, including their origin (analogs or natural). General knowledge of cassava growth and differentiation regulation is scarce.

Histological analyses using fixed, stained, and microtome-processed samples as well as those using free-hand sections of cultured unpollinated ovules suggest that solid F6 and solid BDS media induced embryo formation. No sign of embryo development was noted in any of the samples analyzed from cultures on solid A1 or solid CBM medium. It also appears that F6 apparently promotes a more advanced stage of embryo development than BDS medium during the same period. Data indicate the presence from two-cell to globular embryos on BDS medium, and globular- to torpedo-shaped embryos on F6 medium. In summary, globular embryos were identified in cultures on either BDS or F6 medium, whereas torpedo-shaped embryos were noted only on F6 medium. F6 medium is based on BDS medium micro- and macro-nutrients unlike CBM and A1 media. F6 contains 5 times less thiamine and myo-inositol and it does not contain L-proline in contrast to BDS medium. Further studies with larger and genotypically variable material should show whether these modifications are essential for gynogenesis induction. The growth regulator levels applied during the study did not differ among applied induction media; thus, this effort is to be made as the next step in gynogenesis studies, considering the importance of the growth regulators in embryo and plant growth and differentiation.

Although careful microscopic analyses were performed for cultures that were incubated on BDS or F6 medium for 6 weeks, free-hand sections were performed on ovules transferred onto R medium for 9 weeks after the initial culture on BDS or F6 medium for 6 weeks. Thus, we conclude that transferring ovules onto R medium allows for a more advanced development of induced embryos to late globular and torpedo shape. However, a larger number of samples needs to be analyzed in order to statistically confirm this hypothesis.

A1 and CBM media induced the lowest gynogenic response. A1 medium is based on B5 medium (Gamborg et al., 1968). CBM medium is used for gynogenic induction in cucumber (Gémes-Juhász et al., 2002). The BDS (Bohanec and Jakse, 1999), F6, and R media (Michalik et al., 2000) used in this work are all modifications of the original BDS medium proposed by Dunstan and Short (1977). These media had been used in a broad range of onion varieties that differed in their gynogenic response. Well-documented studies in onion indicate that increased gynogenic response is obtained when using a two-step rather than a one-step protocol (Michalik et al., 2000; Fayos et al., 2015). Furthermore, a two-three times higher percentage of embryogenesis and plant acclimation was attained when flower buds of low-responding Spanish onion genotypes were cultured in a two-step protocol (Fayos et al., 2015). Although the BDS, F6, and R media differed in their composition of vitamins, amino acids, other organic supplements, and growth regulators, it has been suggested that the 2ip and NAA in the R medium, instead of 2,4-D and BAP in BDS and F6 media, could account for the higher gynogenic efficiency (Fayos et al., 2015). Higher percentages of gynogenesis were also reported with Polish onion cultivars, with media containing 2ip and NAA (Michalik et al., 2000). Therefore, our results show effects similar to those described by these authors.

In this work, despite the various factors evaluated, it was not possible to sustain embryo differentiation beyond the globular-torpedo stage and the recovery of fully developed plants. The same difficulty was reported from studies on cassava androgenesis, in which globular mass cell structures obtained *in vitro* did not advance in development (Wang et al., 2011; Perera et al., 2014a, b). It is well documented that the culture medium is one of the main factors affecting gynogenesis induction and the recovery of haploid/DH plants (Dong et al., 2016). However, these requirements are also highly dependent on the genotype and the growing conditions of the donor plants, which affect the vigor and the physiology of the tissues. Cassava is well known for its recalcitrance and high genotype-dependent response to various *in vitro* culture techniques (Chavarriaga-Aguirre et al., 2016). So far, the potential of using biotechnology to improve cassava has been significantly dependent on the success in optimizing and adjusting the various technologies specifically to this crop. Progress and lessons learned from other crops have so far been of limited use. Therefore, it is necessary to study further the culture conditions to optimize them according to nutritional requirements and growth regulator levels suitable for cassava. The asynchrony of embryo development complicates matters even further.

Follow-up studies should focus on determining the best conditions that would allow maturation of immature cassava zygotic embryos and, ultimately, the recovery of plants from cassava carpel cultures of cyathia hand-pollinated with cassava pollen. The results could help in defining the optimal conditions for haploid embryo growth in cassava. The knowledge generated from such studies would help define the optimal conditions inducing gynogenic response in cassava when using several alternative methods (gynogenesis of unpollinated ovules, embryo growth in ovules pollinated with pollen of distant species or with irradiated pollen).

Another major bottleneck found in the current work was the difficulty in documenting the development of embryos in ovary/ovule cultures, information needed to be able to make a science-based decision about the best treatments in cassava. A methodology for histology analysis combining tissue sectioning using vibratome and clearing techniques for documentation of embryo development from the very early stages could also be useful as a tool for mass screening of ovary/ovule cultures.

The famous Chinese proverb says, “A journey of a thousand miles begins with one step.” The first important step in cassava gynogenesis is presented above. DH technologies for other important crops had already been investigated more than a half century ago and they were later optimized by many laboratories around the world; thus, breeding can now benefit from efficient protocols. Considering the importance of cassava, we hope that our work will be continued.

## Data Availability Statement

All datasets generated for this study are included in the article/Supplementary Material.

## Author Contributions

ZL and MW designed the research and wrote the manuscript. ÁG, ET, and MB performed the research under the supervision of ZL and MW. All authors analyzed the data, read and approved the final manuscript.

## Conflict of Interest

The authors declare that the research was conducted in the absence of any commercial or financial relationships that could be construed as a potential conflict of interest.
