# Supplementary material for: Studies on Gynogenesis Induction in Cassava (Manihot esculenta Crantz) Unpollinated Ovule Culture
Source: Front Plant Sci. 2020 Apr 3;11:365. doi: 10.3389/fpls.2020.00365 (PMC7147354; doi:10.3389/fpls.2020.00365)
Supplement: Supplementary file 1 [file Data_Sheet_1.PDF]

Supplement Table 1. Media composition evaluated for gynogenic induction in cassava

Components	Medium				
	CBM[a]	A1[b]	F6[b]	BDS[c]	R[b]
Based on	CBM[a]	B5[d]	BDS[e]	BDS[e]	BDS[e]
Macro- and micro-elements (mg/L)					
KNO_3	950	2500	2530	2530	2530
NH_4 NO_3	450	-	320	320	320
$(NH_4)_2SO_4$	17.5	134	134	134	134
$Ca(NO_3)_2$ x $4H_2O$	25	-	-	-	-
$MgSO_4$ x $7H_2O$	185	249	247	247	247
$NaH_2PO_4.H_20$	19	-	-	-	-
$NaH_2PO_4.2H_2O$	-	170	172	172	172
KH_2PO_3	75	-	-	-	-
KCl	3.5	-	-	-	-
$NH_4H_2PO_4$	-	-	230	230	230
$CaCl_2$	160	-	-	-	-
$CaCl_2$ x $2H_2O$	-	150	150	150	150
$MnSO_4$ x $4H_2O$	-	-	13.2	13.2	13.2
$ZnSO_4$ x H_2O	4	10	-	-	-
$ZnSO_4$ x $7H_2O$	-	-	2	2	2
H_3BO_3	4	2	3	3	3
KI	0.7	3	0,75	0,75	0,75
$CuSO_4$ x $5H_2O$	0.016	0,75	0,039	0,039	0,039
Na_2MoO_4 x H_2O	-	0,025	-	-	-
Na_2MoO_4 x $2H_2O$	0.2	-	0,25	0,25	0,25
$CoCl_2$ x $6H_2O$	0.016	0,025	0,025	0,025	0,025
$FeSO_4$ x $7H_2O$	27.85	27.85	27.85	27.85	27.85
Na_2-EDTA	37.85	37.85	37.85	37.85	37.85

Supplement Table 1. Continued

Components	Medium				
	CBM[a]	A1[b]	F6[b]	BDS[c]	R[b]
Based on	CBM[a]	B5[d]	BDS[e]	BDS[e]	BDS[e]
Vitamins, amino acids, other organic supplements (mg/L)					
Nicotinic acid	1	1	1	1	1
pyridoxine	2	1	1	1	1
thiamine	1	2	2	10	2
glycine	0.1	2	-	-	-
folic acid	1	1	-	-	-
Ca-pantothenate	0.5	1	1	-	1
Biotin	0.05	0.01	-	-	-
L-proline	-	-	-	200	200
adenine	-	-	-	-	10
Myo-inositol	80	100	100	500	500
Sucrose	100000		100000	100000	100000
2,4-D	2	-	2	2	-
NAA	-	-	-	-	1
BAP	2	-	2	2	-
2ip	-	-	-	-	2
Gelrite	4 g				

[a] Gémes-Juhász et al. (2002)
[b] Michalik et al. (2000)
[c] Bohanec, B., and Jakse, M. (1999)
[d] Gamborg et al. (1968)
[e] Dunstan and Short (1977)
